# Twinning-induced plasticity (TWIP) and work hardening in Ti-based metallic glass matrix composites

**DOI:** 10.1038/s41598-017-02100-9

**Published:** 2017-05-12

**Authors:** J. Fan, J. W. Qiao, Z. H. Wang, W. Rao, G. Z. Kang

**Affiliations:** 1 Laboratory of Applied Physics and Mechanics of Advanced Materials, College of Materials Science and Engineering, Taiyuan University of Technology, Taiyuan, 030024 China; 2 Key Laboratory of Interface Science and Engineering in Advanced Materials, Ministry of Education, Taiyuan University of Technology, Taiyuan, 030024 China; 3 Shanxi Key Laboratory of Material Strength and Structural Impact, Institute of Applied Mechanics and Biomedical Engineering, Taiyuan University of Technology, Taiyuan, 030024 China; 4 Key Laboratory of Advanced Technologies of Materials, School of Mechanics and Engineering, Southwest Jiaotong University, Ministry of Education of China, Chengdu, Sichuan 610031 China

## Abstract

The present study demonstrates that Ti-based metallic glass matrix composites (MGMCs) with a normal composition of Ti_43_Zr_32_Ni_6_Ta_5_Be_14_ containing ductile dendrites dispersed in the glass matrix has been developed, and deformation mechanisms about the tensile property have been investigated by focusing on twinning-induced plasticity (TWIP) effect. The Ti-based MGMC has excellent tensile properties and pronounced tensile work-hardening capacity, with a yield strength of 1100 MPa and homogeneous elongation of 4%. The distinguished strain hardening is ascribed to the formation of deformation twinning within the dendrites. Twinning generated in the dendrites works as an obstacle for the rapid propagation of shear bands, and then, the localized necking is avoided, which ensures the ductility of such kinds of composites. Besides, a finite-element model (FEM) has been established to explain the TWIP effect which brings out a work-hardening behavior in the present MGMC instead of a localized strain concentration. According to the plasticity theory of traditional crystal materials and some new alloys, TWIP effect is mainly controlled by stacking fault energy (SFE), which has been analyzed intensively in the present MGMC.

## Introduction

Bulk metallic glasses (BMGs) are recognized as potential structural materials due to their superior performance, such as high strength, large elastic limit, and excellent corrosion and wear resistance, etc.^1^. However, they generally fail in a brittle manner under uniaxial quasistatic loading at room temperature^2^. To alleviate this challenge, a series of *in-situ* dendrite-reinforced metallic glass matrix composites (MGMCs) with large tensile ductility have been developed, such as Ti-based and Zr-based MGMCs^3–5^. However, most of these composites exhibit softening rather than work hardening after yielding upon tension at room temperature, associated with localized necking. In contrast, generally, the traditional crystalline alloys such as steels, Ti as well as Al alloys^6–8^, and new promising crystalline alloys such as high-entropy alloys (HEAs)^9^, have outstanding tensile work-hardening capacity. And the excellent properties are induced by transformation-induced plasticity (TRIP) and twinning-induced plasticity (TWIP) effects, which is closely related to stacking fault energy (SFE)^10^. Referring to MGMCs, up to now, the work-hardening capability mainly relies on the micro-mechanisms of TRIP effect, including the martensite transformation in CuZr-based MGMCs^11–13^, and the transformation from β to α phase in Ti-based MGMCs^14, 15^. However, for most *in-situ* MGMCs including thermally stable dendrite-reinforced composites, the work-hardening is rarely available^4^, unless the volume fraction of stable secondary phases takes value very high so that a network and even disconnected structures of amorphous phases form^16^. In other words, the irreversible phase transformation favors the availability of work hardening in MGMCs after yielding, which has been the sole way to acquire the flow stress larger than yielding stress so far.

In this work, a novel Ti-based MGMCs with macroscopic tensile ductility and work-hardening capacity has been developed. The composites consist of the amorphous matrix and β dendrites with a metastable body-centered cubic (bcc) structure as the secondary phases. Upon loading, the shear bands generated within the glass matrix can be arrested by the ductile dendrites, which avoids catastrophic rupture with highly-localized shear banding and results in excellent fracture toughness. It is noted that the work-hardening behavior induced by the deformation twins formed within bcc dendrites is firstly reported here. And, almost no phase transformation happens within dendrites during plastic flows. Usually, twinning frequently occurs in the TWIP steels, which is related to SFE. When the slip is impeded during plastic deformation, a new slip system would be activated by twinning, leading to a strengthening effect. Thus, the deformation twins can remarkably improve the tensile strength^6, 10, 17^. The present composite with secondary dendrites not only settles the softening shortcoming appearing in the common MGMCs, but also guarantees the high yielding strength. By investigating mechanical responses under tension, the deformation mechanisms in this study are emphasized on the TWIP effect in β dendrites.

## Experiment

Master alloys with a normal composition of Ti_43_Zr_32_Ni_6_Ta_5_Be_14_ were produced by arc melting the mixture of pure elements Ti, Zr, Ni, Ta, and Be with purities greater than 99.9% (wt.%) under a Ti-gettered argon atmosphere. Plate-shape samples with 2 mm in thickness and 10 mm in width were obtained by the copper-mould-casting method. Dog-bone-like plate specimens with gauge dimensions of 10 mm (length) × 2 mm (width) × 2 mm (thickness) were well polished and used for tensile tests. The tension was conducted using a strain rate of 5 × 10^−4^ s^−1^. The as-cast and fractured samples were observed by scanning-electronic microscope (SEM) to examine the microstructure and fractographs. Crystalline phases were identified by X-ray diffraction (XRD), transmission electron microscopy (TEM), and high resolution TEM (HRTEM) in a JEM-2010 microscope. The specimens for TEM were prepared by mechanical grinding followed by ion milling. A Nano Indenter II tester (MTS Systems, USA) with a trihedral Berkovich indenter was used to calculate the Young’s modulus of both the glass matrix and dendrites at room temperature.

## Results

**Figure 1:**
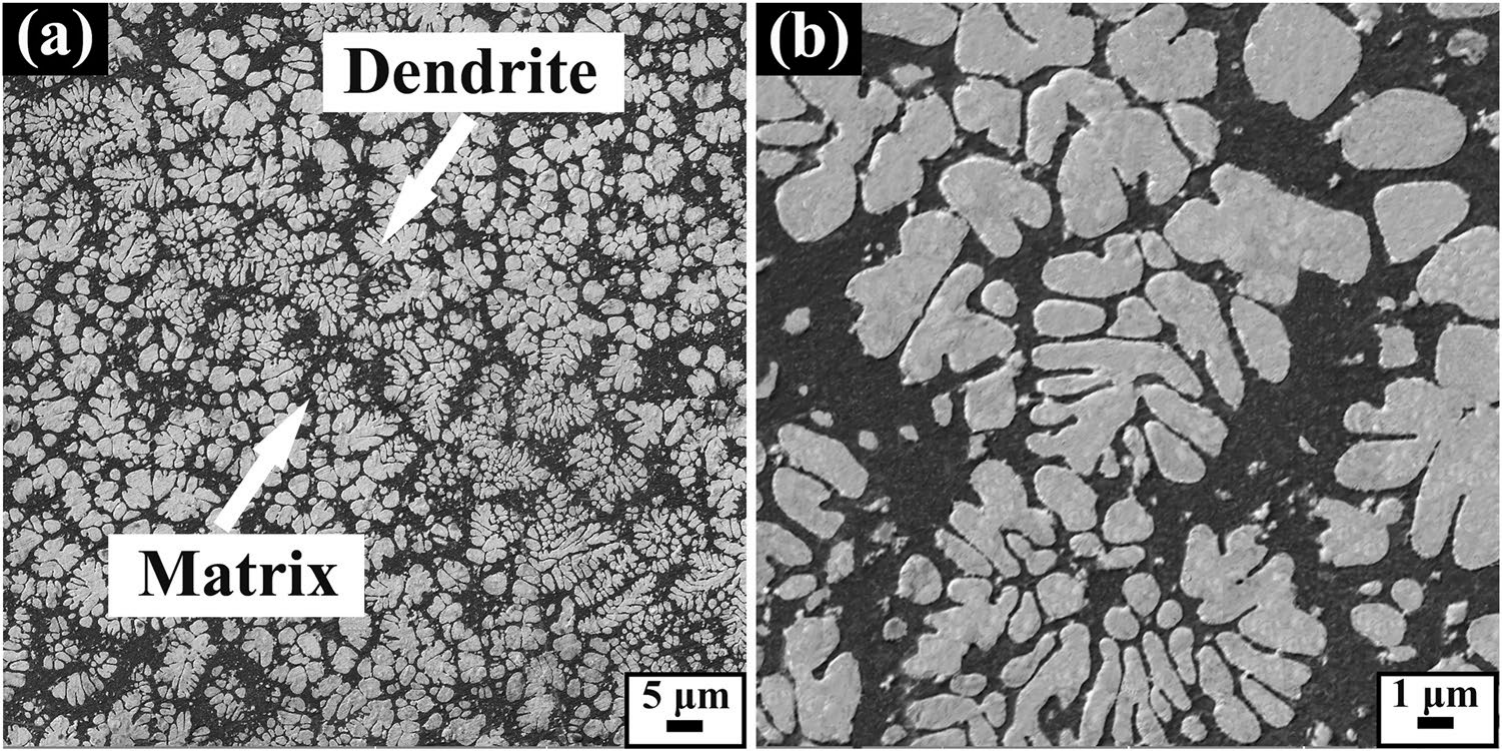
(**a**) Low and (**b**) high magnification SEM images of the present composites.

Figure 1(a) shows the microstructure of the as-cast Ti_43_Zr_32_Ni_6_Ta_5_Be_14_ MGMCs. It can be seen that *in-situ* precipitated dendritic phase is homogenously embedded in the amorphous matrix. The volume fraction of the dendrites takes a value of approximately 54%, and the analysis of EDS gives an average composition of the glass matrix and dendrites, which are characterized as Ti_35.7_Zr_38.3_Ta_4.0_Ni_8.0_Be_14_ and Ti_50.3_Zr_37_Ta_8.6_Ni_4.1_, respectively. The element Be is considered to only exist in the matrix totally^3, 4^. The spacing of primary dendrites is about 0.5–1 μm, determined from a higher magnification of the SEM image, as displayed in Fig. 1(b).

**Figure 2:**
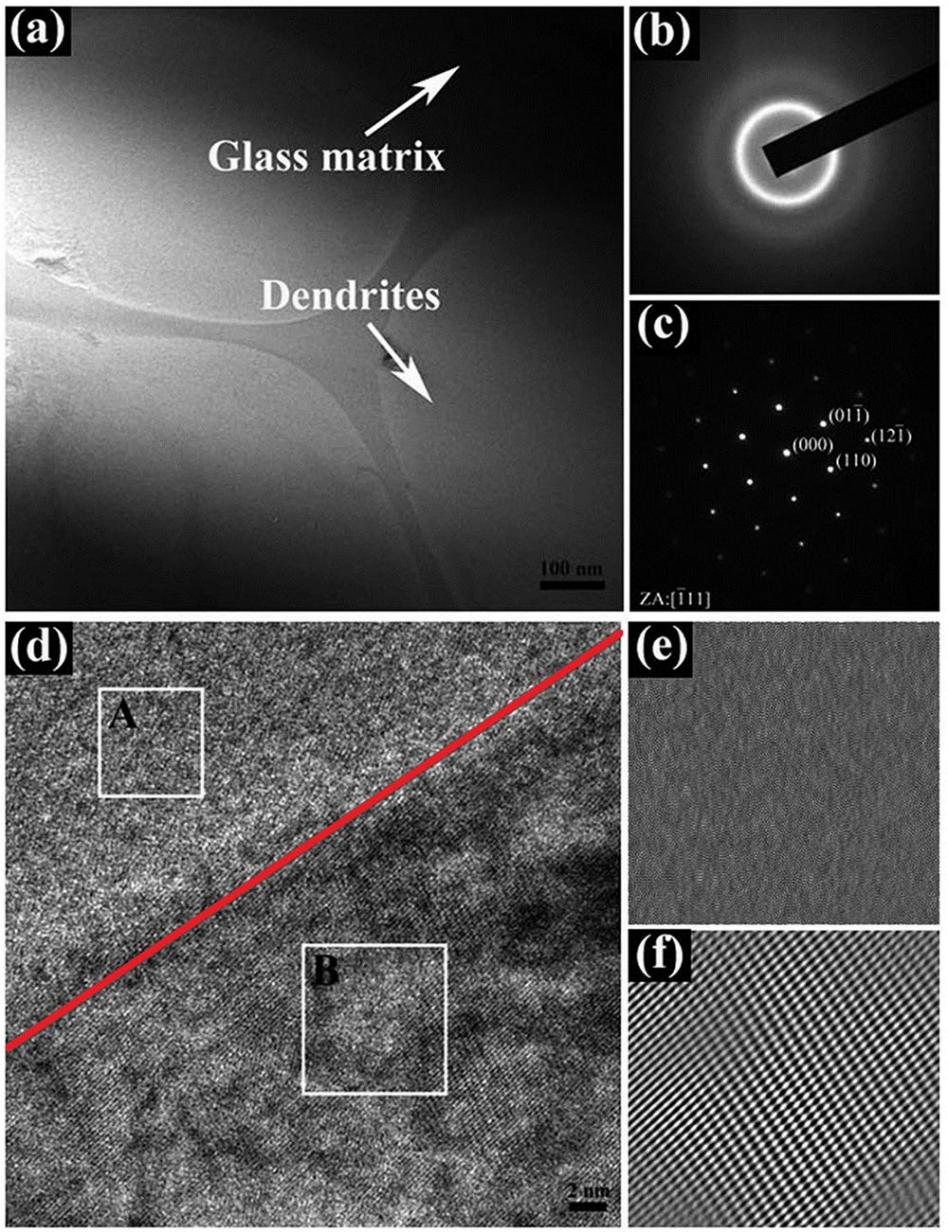
(**a**) BF TEM image of the present Ti-based MGMCs at low magnification; SAED patterns of (**b**) the matrix and (**c**) the dendrites; (**d**) HRTEM image taken near the interface; IFFT patterns of (**e**) the glass matrix and (**f**) the dendrites, marked by rectangles in (**d**), near the interface are shown in A and B, respectively.

To further exactly identify the dual-phase structure, the TEM technique has been employed to analyze the structure of the as-cast composites. A bright field (BF) TEM image at low magnification is displayed in Fig. 2(a). The light and dark areas, denoted by the arrows, indicate the dendrites and glass matrix, respectively. Figure 2(b) and (c) are the selected area electron diffraction (SAED) patterns of the matrix and dendrites, respectively, which further identifies the amorphous nature of glass matrix with only diffuse halos and the bcc structure of β-Ti dendrites with a lattice parameter of a = 0.3319 nm in the zone axis (ZA) of $[\bar{1}11]$. An HRTEM image near the boundary between the matrix and dendrites is clearly shown in Fig. 2(d) (as depicted in red line). What can be seen is that the atomic bonding between two phases has been proven to be very good, and an atomically-sharp interface is well confirmed. Figure 2(e) and (f) are inverse fast Fourier transform (IFFT) patterns of the glass matrix and dendrites, respectively, corresponding to the areas marked by rectangles in Fig. 2(d) (A and B represent the matrix and crystalline phases, respectively). Obviously, lattice defects such as dislocations in the dendrites are rarely found. Similar results have been demonstrated in other *in-situ* dendrite/MGMCs^3, 4^. Instead, a regular arrangement of lattice patterns prevails, and no ordered structure is available within the glass matrix.

**Figure 3:**
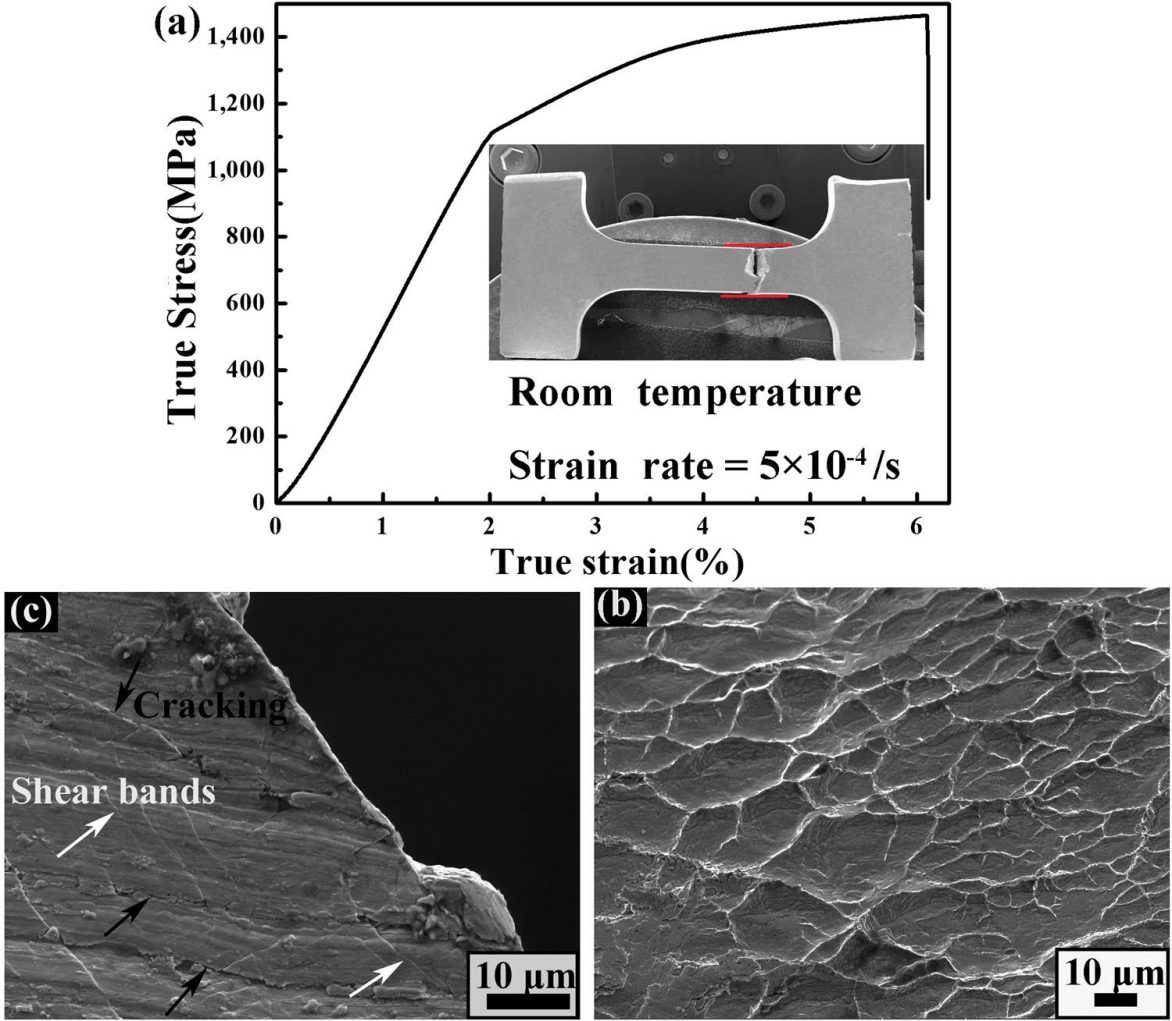
(**a**) Tensile true stress-strain curve of the present Ti-based MGMCs, the inset showing a low-magnification SEM image of the fractured sample and the strain rate of 5 × 10^−4^ s^−1^; (**b**) Vein patterns showed on fracture surface; and (**c**) the lateral surface after tensile deformation, demonstrating the shear bands and significant cracking, indicated by arrows.

Figure 3(a) exhibits a typical true stress–strain curve of the present composite upon tension at ambient temperature. The tensile tests are conducted more than 3 times in order to well confirm the results. As can be seen, the present composite yields at ~1100 MPa, followed by a significant work hardening. After achieving an ultimate tensile strength of ~1470 MPa, the flow stress stops increasing, and the strain gets to the extreme. Consequently, the final fracture occurs at the moment. On average, the total tensile ductility of the current MGMCs reaches about 4%. The low-magnification image of the fractured specimen is presented in the inset of Fig. 3(a). The width of the gauge, indicated by the red lines, almost keeps homogeneous, which means a homogeneous elongation without necking in the whole specimen upon tension. After fracture, a vein pattern, the characteristic of typical fracture in BMGs, can be observed in Fig. 3(b). Meanwhile, the lateral surface of the fractured sample is shown in Fig. 3(c). Profuse primary shear bands parallel to the fracture surface and obvious cracks near the fracture surface are found, as denoted by the bright and dark arrows, respectively.

**Figure 4:**
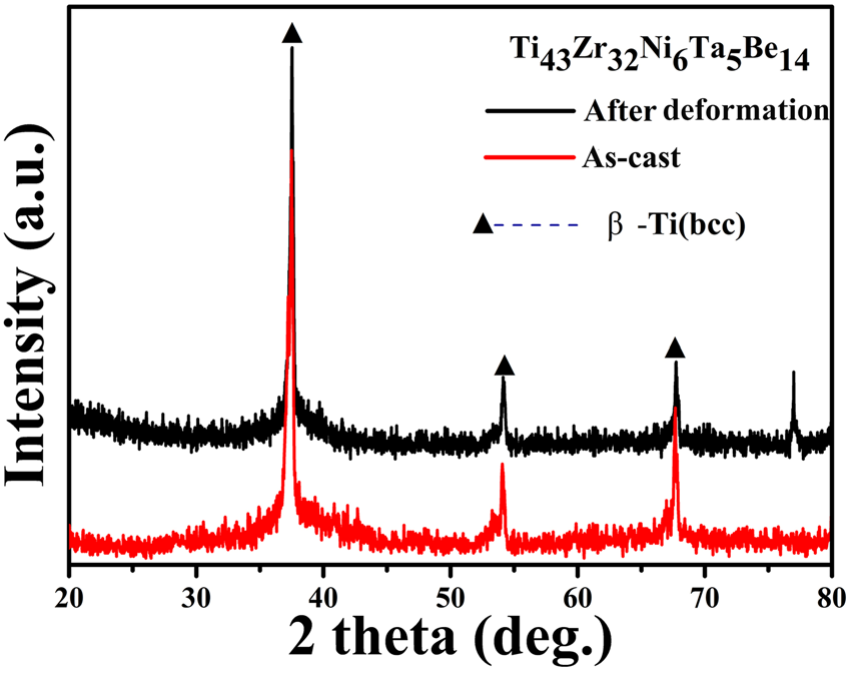
XRD patterns of the present Ti-based MGMCs before and after tension.

XRD has been conducted on the as-cast and deformed samples to clarify the structure of the composites, as displayed in Fig. 4. The sharp crystalline peaks superimposed on the broad diffuse diffraction maximum prove that the composites consists of bcc β-Ti and amorphous phases. Compared with XRD patterns taken from the samples before and after tension, there exists no essential difference between them, which may further confirms that no phase transformation occurs during work hardening. Such kind of deformation without any phase transformation happens in most *in-situ* dendrite-reinforced MGMCs reported^18^.

**Figure 5:**
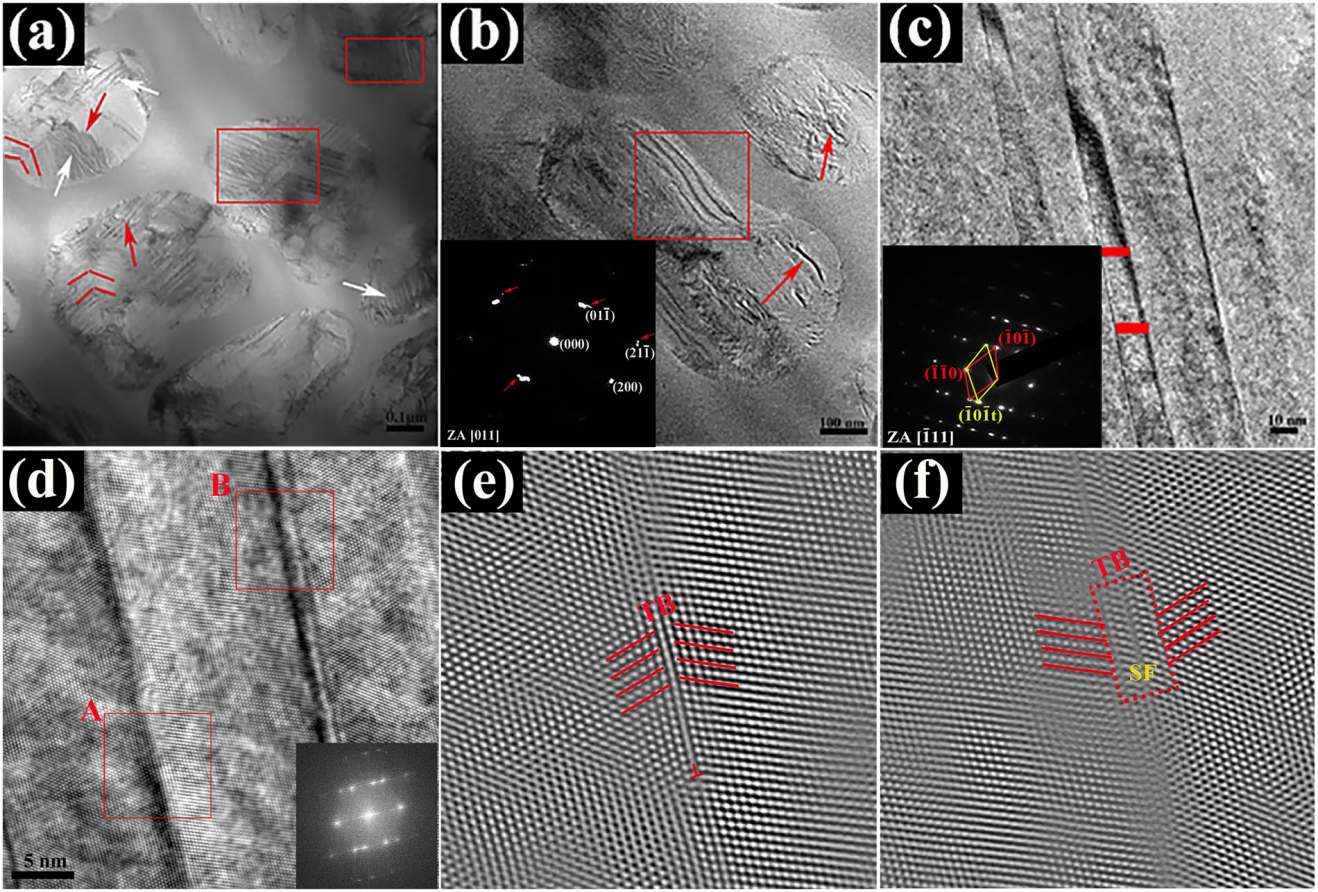
(**a**) BF TEM image of the present Ti-based MGMCs after deformation; (**b**) the magnified TEM image of special deformed dendrites, the inset corresponding SAED pattern; (**c**) the magnified TEM image of the twin structures in dendrites and the insert image corresponding SAED pattern; (**d**) The HRTEM image of the twin structures and the FFT pattern shown in inset; (**e**) and (**f**) are IFFT images of the part of rectangle region A and B in (**d**), respectively.

Although XRD results characterize most macroscopic situations within samples, more details should be focused on microscopic scale to unveil the deformation mechanisms. Figure 5 reveals detailed microscopic images of the composites after tension. A BF TEM image of the deformed sample is shown in Fig. 5(a). Compared with the undeformed sample shown in Fig. 2(a), the severe fragmentation of dendrites is dominant after tension, and a number of deformation bands with different orientations occur within dendrites. Analogous fragmentations are essentially found in stable dendrites only if the phase transformation is not accompanied^4^. As can be seen, parallel deformation bands and symmetrical deformation zones are substantially found in dendrites, denoted by the bright arrows and red lines, respectively. Apparently, the deformation bands are identified to be twinning by TEM analysis. The areas, marked with the red rectangle in Fig. 5(a), demonstrate the existence of a large number of stacking faults within dendrites, which would evolve into twinning.

Figure 5(b) presents the magnified image of seriously deformed dendrites, among which the twinning bands and microcracks are observed, denoted by the rectangle and arrows, respectively. The SAED pattern shown in the inset of Fig. 5(b) indicates severe lattice distortion, and a great deal of SFs occur within dendrites, which can be observed from the shift of spots, denoted by the red arrows^19^. Figure 5(c) shows a larger magnified image of the deformation twins, and the corresponding SAED pattern inserted in the figure identifies the structure of the deformation twinning. What’s more, it can be observed that the average width of the twinning is about 10 nm, denoted by the red line, agreeing with the width found in CuZr-based MGMCs^11, 12^. Figure 5(d) exhibits the HRTEM image of the twining, and the corresponding FFT image is shown in the inset. The IFFT image in Fig. 5(e) is taken from the part of rectangle A in Fig. 5(d), from which the feature of the deformation twins is clearly visualized, as denoted by the red line, and visible dislocations at the grain boundary of the twinning can be found. Figure 5(f) shows the IFFT image corresponding to the part of rectangle B, the deformation twinning at the twinning boundary is displayed, as denoted by the red line. And stacking faults can be observed at the twinning boundary, as shown in the part of dash rectangle in Fig. 5(f). Twinning is a fundamental deformation mode that competes against dislocation slip in crystalline alloys. Generally, higher stress is thought to favor twinning over the dislocation slip, and deformation twinning has been well documented in fcc and hexagonal-close packed (hcp) alloys^20^. Scarcely, twinning is essentially found in nano- and micro-scaled bcc crystalline alloys such as Ta^21^, W^22^, Mo^23^, and V crystals^24^. Previously, in *in-situ* MGMCs, the dendrite phase is mainly composed of Zr-Ti-Nb (V). Accordingly, the dislocation slip rather than twinning is the dominating deformation mode. In the current composite, Ta-rich bcc solution may favor the availability of twinning during plastic flows. Ogata *et al*.^25^ have proposed that the variation of SFE has its origins from the electronic structures, and closely relates to the charge density change during the stacking fault formation. According to this criterion, Wu *et al*. obtained that Ta has very high SFE compared with Co, Ni, Cr, Ag, Ti, and Hf^26^. However, twinning easily generates in the micro-scaled and nano-scaled Ta crystalline samples during plastic deformation^27^. And the bulk samples are tested in this study, discontinuous network structure in micrometer-sized dendrites including element Ta is predominant, which facilitates twinning happening in deformed dendrites.

## Discussion

**Figure 6:**
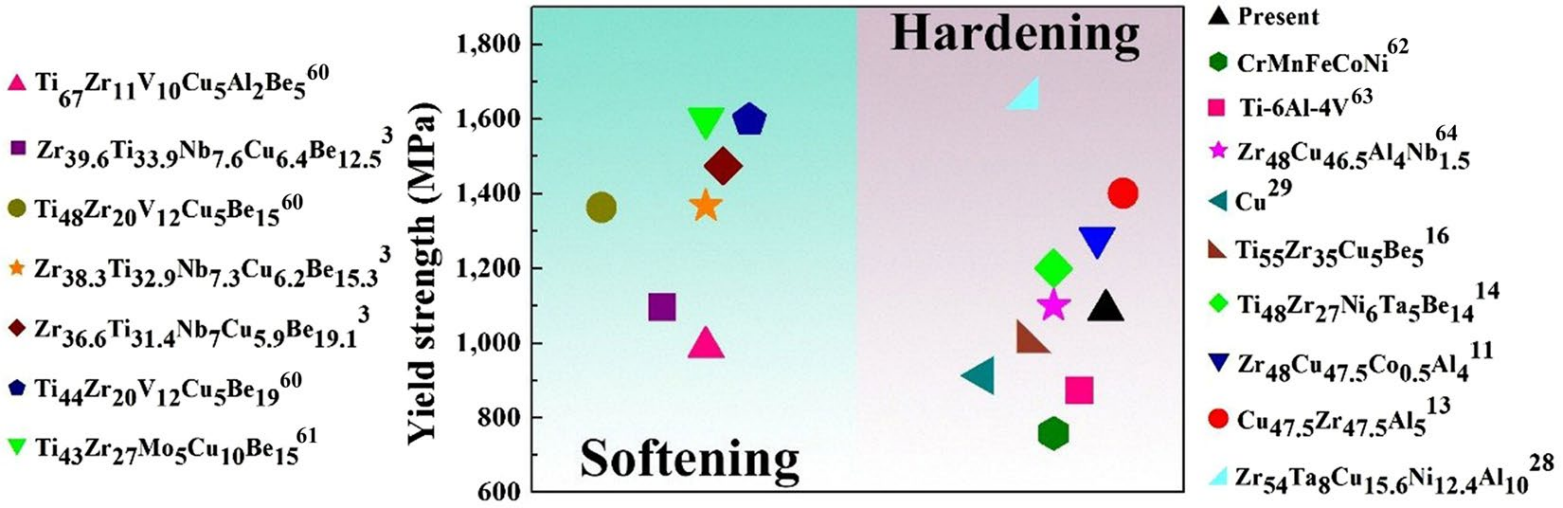
Alloys with different deformation behaviors upon tension.

The deformation behavior in MGMCs containing ductile dendrites under tensile loading is attributed to the unique plastic deformation mechanisms. The deformation mechanisms in all kinds of *in-situ* MGMCs with a macroscopic scale exhibit softening or hardening after yielding upon tensile loading. Softening is usually induced by the multiplication of shear bands in the glass matrix, while hardening can appear when the TRIP or TWIP effect reacts within the composites. Figure 6 summarizes mechanical behaviors of different alloys that covers MGMCs, together with traditional and new metals and alloys such as HEAs, which exhibit either softening or hardening behaviors after yielding upon tension. The flow stress (*σ*_*f*_) during plastic deformation, corresponding to the hardening behavior, is larger than yielding stress (*σ*_*y*_). And, an opposite case occurs with softening behavior. Here, different from the ductilization mechanisms induced by phase transformation and/or typical particles such as Ta within MGMCs^28^, the main plastic deformation mechanisms in the current composites are ascribed to deformation twins. Twins can be formed through various approaches including plastic deformation at extremely low temperature and/or high strain rate, phase transformation, thermal treatment, and other physical or chemical processes in a large variety of metals and alloys^29, 30^. Dipankar *et al*. and Sun *et al*. have found that TRIP and TWIP effects can occur in the Ti alloys, which contains a bcc phase^31, 32^. While, nanoscaled mechanical twins are not easily achieved experimentally under usual deformation conditions in MGMCs^33^. Neither with an unique structure nor through special processing, the deformation twinning ensures high yield strength and outstanding work-hardening capacity in the present composite. It means that the understanding about deformation mechanisms in MGMCs under tension is limited, and the TWIP effect in MGMCs is discussed later.

The studies about the TWIP effect are mainly focused on the TWIP steels^34, 35^, and the strain-hardening capacity is controlled by the mechanical twins. The deformation twins induced by high tensile stress exert influences on mechanical properties by acting as the effective obstacles for dislocation movement according to the Hall-Petch type strengthening mechanisms. The local deformation is hindered, and the necking phenomenon may be avoided^34–36^. It is well known that shear bands are initiated firstly in the amorphous matrix, and then, the ductile dendrites are deformed to hold back the generation of multiple shear bands^14^. This leads to intense interaction of interface between the matrix and dendrites, which causes a remarkable change in the interfacial transition zone^35^. It is noted that the shear bands generated in the matrix are concentrated at the boundary, caused by the stress concentration at the interface. And the dendrites that are subject to the high stress easily produce a large amount of dislocations to hinder the propagation of shear bands^4, 5, 11^. Different from the dislocation activity mechanisms, the deformation twinning here determines the deformation mechanisms. Wang *et al*. has found the formation of deformation twinning in nanocrystalline Ta^21^. Oh *et al*. reported the TWIP effect in Ti alloys containing Ta^14^, which means that Ta is beneficial for the formation of deformation twinning. From Fig. 5(e) and (f), the dislocations and stacking faults at the twinning boundary can be identified. The dislocations movement here is blocked firstly, and then, the dislocations dissociate into partial dislocations, which is a favorable process to produce the stacking faults. A lot of stacking faults superpose to form the deformation twinning, which is displayed in Fig. 5(f). At the twinning boundary, there are many stacking faults and partial dislocations. While, the twinning boundary in Fig. 5(e), displays a smooth boundary, indicates that the evolution from stacking faults to deformation twins is a process of the nucleation and growth of the twins^29, 35, 37^. More significantly, the stress concentration is another crucial factor for the generation of deformation twinning. As shown in Fig. 5(a), the deformation twinning is mostly distributed at the edge of dendrites, in agreement with the stress concentrating at the interface. It reveals that the high strength of the matrix provides critical stress to generate twins. Along with the formation of profuse twins, the TWIP effect becomes dominating mechanisms to accommodate plasticity^38^. And the deformation twinning could stop the propagation of shear bands, and meanwhile prevent the movement of dislocations. Therefore, the current composite exhibits work-hardening behavior under tensile loading. On the other hand, the deformation twins in crystalline dendrites with different orientations can be considered as the sub-boundaries, which results in high strength in the present composite^39^. The twins with different orientations penetrate dendrites from the boundary to inside, which are followed by the formation of more twins with increasing the stress. The size of the twinning keeps in nano scale, as displayed in Fig. 5(b), which makes contribution to excellent properties, referring to the work of Lu *et al*.^29^. Furthermore, the growth of cracks can be effectively blunt by twins, and the deformation twinning among dendrites prevents the formation of individual mature shear bands. Instead, profuse intersectant shear bands prevail, indicative of distinguished plasticity, which is consistent with fractographs shown in Fig. 3(c). As a result, a homogenous elongation of such a kind of dual-phase composites appears (Figure 3(a))^40^. As mentioned above, the stress concentration in the deformed dendrites induces twins. The nucleation and growth of deformation twins among dendrites can absorb much strain energy, which inhabits the multiplication of shear bands in the glass matrix associated with softening. The dendrites and deformation twins could interrupt the propagation of shear bands, which avoids the catastrophic rupture. The conclusion can be drawn that the deformation twins in dendrites, together with multiple shear bands in the glass matrix, ensure the high strength as well as good ductility in such a kind of composites.

**Figure 7:**
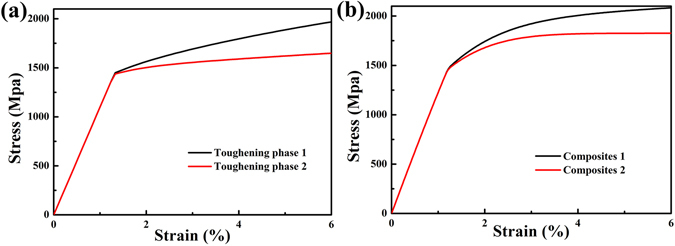
The tensile stress-strain curves from the finite-element model, Fig. 7(a) shows the tensile stress-strain curves for different toughing *in-situ* dendrites within MGMCs, the black one responses to a work hardening behavior induced by deformation twining and the other one (red one) has softening without TWIP effect. The black curve in Fig. 7(b) represents the composite consisted by toughing phase 1 in Fig. 7(a), and the red curve correspond to the toughing phase 2 in Fig. 7(a).

**Figure 8:**
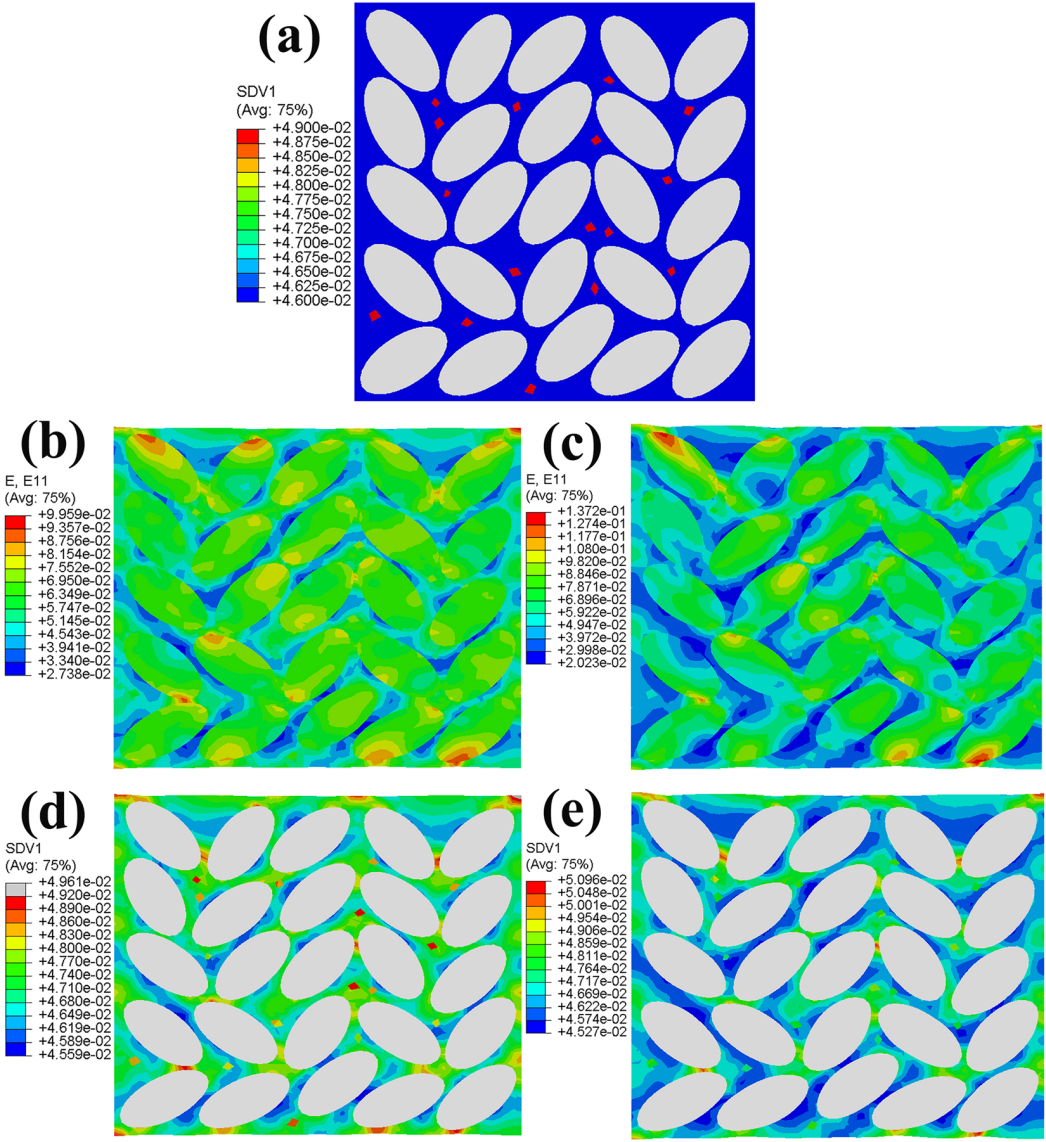
(**a**) The initial state of the composites in FEM, and the maps of strain contours for composite 1 and 2 correspond to Fig. 7(b) with a tensile strain of 6% are shown in (**b**) and (**c**), respectively. (**d**) and (**e**) represent the evolution of shears bands of two different MGMCs.

The ductility of MGMCs is closely related to the stress distribution between the matrix and dendrites upon tension. The *in-situ* dendrites influence the stress distribution within the composites, and lead to a softening or hardening behavior under tension. As required, the deformation behaviors of MGMCs are described by the free-volume and elastic-plastic models, which are incorporated into a finite-element code (ABAQUS) as a user material subroutine^41^. And the concentration of free volumes, which can be used to characterize the shear bands, is denoted as an internal state variable of SDV1 in ABAQUS. The TWIP effect benefits to the work hardening occurrence as shown in experimental results^29^. And in order to explain the evolution of shear bands, the multilinear elastic-plastic model is established. The present MGMCs with obvious work-hardening capacity is characterized by the TWIP effect. Guided by the work of Jiang *et al*.^42^, the 2-D plane-strain finite-element model (FEM) is developed in this work. Within this model, the loading boundary is coupled, and the displacement loading, which is perpendicular to the loading boundary, is applied at the corresponding reference set. The opposite boundary to the loading boundary is applied to the normal constraints, and a vertex belongs to the opposite boundary is applied to full constraints. It should be noted that although the tough particles such as dendrites are randomly dispersed in the real composites, a regular distribution of elliptic particles still reflects the main characteristics of the composites to some extent. Thus, the dendrites are assumed to be elliptic particles. The material parameters of the present MGMCs are listed in Table 1 ^41^. Figure 7 is a model based on the stress-strain curves with different *in-situ* dendrites. Figure 7(a) shows the tensile stress-strain curves of different *in-situ* ductile dendrites within MGMCs, in which the black one responses to a work hardening behavior induced by deformation twinning and the other one (red one) shows softening without TWIP effect. The black curve in Fig. 7(b) represents the composite consisting of tough phase 1 in Fig. 7(a), and the red curve corresponds to the tough phase 2 in Fig. 7(a).The comparative explanation of the stress distribution and shear band evolution between the two composites is demonstrated in Fig. 8. Figure 8(a) shows the initial high concentration of free volumes within the glass matrix. The bright ovals represent the dendrites, and the blue areas are the matrix. The red points among the glass matrix indicate the region where free volumes gather, which could subsequently induce local shearing^43^. The maps of strain contours with a tensile strain of 6% are shown in Fig. 8(b) and (c), respectively. Comparing these two maps, the strain distribution in Fig. 8(b), which corresponds to composite 1 in Fig. 7(b), is more homogeneous than that of Fig. 8(c), which conforms to composite 2 in Fig. 7(b). Furthermore, Fig. 8(d), corresponding to Fig. 8(b) and (e), matching with Fig. 8(c), demonstrate the evolution of shear bands. The shear bands within dendrites with TWIP effect in Fig. 8(d) show a uniform development, and even no mature shear bands appear. The deformation twining ensures an apparent work hardening in the MGMCs. The finite-element model announces that secondary dendrites in MGMCs play different roles as the diverse deformation mechanisms. The dendrites within the matrix change the stress distribution, which hinders fast propagation of shear bands. As a result, a macroscopic tensile ductility is available in MGMCs. And the TWIP effect in the current composite refrain from the localized strain concentration, which is necessary to bring out a work hardening, i.e., a homogeneous plastic deformation, under tensile loading.

**Table 1:** Material parameters for MGMC^46^.

*E* = 138 GPa	*v* = 0.36	*α* = 0.8	*ξ*_0_ = 0.046	*χ* = 1.11	*v* ^*^ = 20 Å,
*T* = 300 K	${t}_{0}^{-1}={\nu }_{0}\,\exp (\frac{-{\rm{\Delta }}G}{{k}_{B}T})=324\,{s}^{-1}$			${\tau }_{0}=\frac{2{k}_{B}T}{{\rm{\Omega }}}=414\,{\rm{MPa}}$

**Figure 9:**
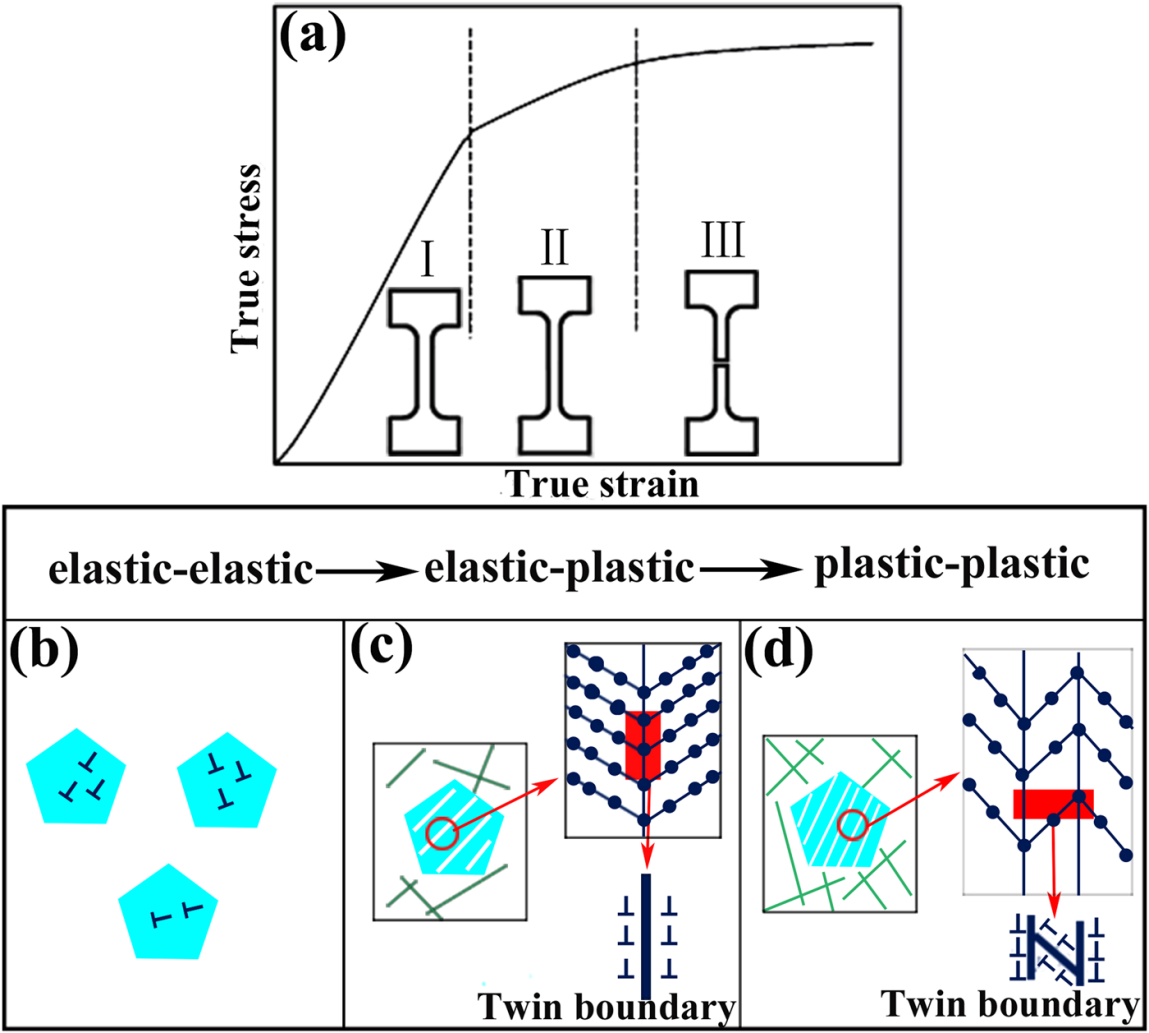
(**a**) True stress-strain curve illustrating the deformation behavior of the present composites, and the corresponding changes during deformation shown in inset. The deformation processes (**b**), (**c**), and (**d**) in the three stages corresponding to the (I), (II), and (III) in Fig. 9(a).

The above detailed deformation mechanisms have been demonstrated by visualized experiments and a simple FEM. Meanwhile, it is of importance to relate with the mathematically theoretical foundation. According to the stress–strain curve shown in Fig. 3(a), the tensile deformation behavior in MGMCs is divided into three stages: (I) elastic-elastic, (II) elastic-plastic, and (III) plastic-plastic, as illustrated in Fig. 9(a) ^44^. The deformation at different stages is schematically illustrated in the inset of Fig. 9(a). In stage (I), elastic–elastic stage, the dual phases are both elastic, and the composite is also elastic as a consequence. The out of sync arises between the dendrites and glass matrix begins in stage (II), elastic-plastic stage. In other words, either dendrites or glass matrix will enter the plastic range, while the other will remain in the elastic range. In this stage, the composite yields and enters into the plastic range. With a continuous increase of the tensile stress, the stage (III), plastic-plastic stage, is approaching, and both the dendrites and matrix in this stage could be plasticity carriers. Therefore, the composite is plastic as well. Referring to the sketch maps from Fig. 9(b) and (d), the changes of deformation structures of the composite in different stages are schematically depicted. As can be clearly seen, the dislocations begin in elastic range while the glass matrix remains unchanged. Concerning the second stage, shear bands within the matrix and deformation twins surrounded by dislocations come into being, which matches the initial work hardening, as shown in Fig. 3(a). The subsequent stage with pile-ups of dislocations and profuse deformation twinning among dendrites indicates successive strain hardening until a final fracture.

According to the above analysis, the stress states of the matrix and dendrites are variant at different stages. Under the circumstance of both phases being elastic, the stress-strain relations for the composites on the basis of Hooke’s law are:

1 $$\begin{array}{cc}\sigma ^{\prime} ={\rm{E}}{\rm{\varepsilon }} & {\rm{\varepsilon }}\le {{\rm{\varepsilon }}}_{{\rm{y}}}({\rm{MPa}})\end{array}$$

where *σ*′, *E*, *ε*, and *ε*_*y*_ are the elastic stress, Young’s modulus, elastic strain, and yielding strain of the composites, respectively. Both of the dendrites and glass matrix being elastic are suitable for Eq. (1).

The Young’s modulus of the composites, *E*_*c*_, can be estimated according to Hashin and Shtrikman^45^.

2 $${E}_{c}={E}_{m}[1+\frac{{f}_{v}({E}_{d}-{E}_{m})}{(1-{f}_{v})\beta ({E}_{d}-{E}_{m})+{E}_{m}}]$$

where *E*_*m*_ and *E*_*d*_ are Young’s moduli of the glass matrix and dendrites, respectively, *f*_*v*_ is the volume fraction of dendrites with a value of 0.538, *β* is the material constant calculated by $\frac{8-10{v}_{m}}{15(1-{v}_{m})}$, and *v*_*m*_ is the Poisson ratio of the glass matrix with a value of about 0.3^2^. *E*_*m*_ and *E*_*d*_ are measured by the nano-indentation to be 136.2, and 92.1 GPa, respectively. The *E*_*c*_ is correspondingly calculated to be 88.7 GPa. At the end of stage (I), the composite starts to be yielding with increasing strain. However, the dendrites and glass matrix yield asynchronously, and the dendrites yield firstly^46^, which means the elastic-plastic deformation begins at the second stage. Meanwhile, the stress can be expressed as follows^47^:

3 $$\sigma =\frac{1}{\sqrt{3}{c}_{d}}[\sqrt{3}{\sigma }_{d}+3{E}_{m}(1-\beta )\frac{1-{f}_{v}}{{f}_{v}}\frac{{\varepsilon }^{p}}{\sqrt{3}}]$$

where *σ* and *σ*_*d*_ are the tensile stress of the composites and dendrites, respectively. *ε*_*p*_ is the plastic strain given as ${\varepsilon }_{p}={f}_{v}{c}_{d}{\varepsilon }_{d}^{p}$ ^47^, ${\varepsilon }_{d}^{p}$ is the plastic strain of dendrites, and *c*_*d*_ is the average stress concentration factor of dendrites, which has a value of 1^48^. Consequently, Eq. (3) can be written in another modality:

4 $$\sigma ={\sigma }_{d}+0.45{E}_{m}{\varepsilon }_{d}^{p}$$

The stress relation between the composites and dendrites is shown in Eq. (4), and the stress impacted on the dendrites can be calculated. It is likely to form the deformation twinning when the stress reaches the critical value. And the critical stress of twinning can be determined by SFE, as reported in typical fcc metals^49, 50^. The stacking faults are produced due to the rearrangement of atoms, which influences the slip and could be considered as a precursor for the formation of twinning in dendrites. The critical shear stress (*τ*_*SF*_) for the formation of the stacking fault is expressed as follows^51^:

5 $${\tau }_{SF}=\frac{\gamma }{b}$$

where *γ* is the SFE, and *b* is the Burgers vector of the perfect dislocations. And the critical shear twinning stress (*τ*_*T*_) is calculated to be as follows^52^:

6 $${\tau }_{T}=\frac{2\gamma }{{b}_{p}}$$

where *b*_*p*_ is the Burgers vector of the partial dislocations. Concerning the critical shear stress (*τ*), a relationship based on the equation of *τ* = *σ*_*n*_ *m* is established. Here, *σ*_*n*_ and *m* represent the normal stress and Schmid factor, respectively. The maximum Schmid factor for deformation twins is 0.5. Therefore, the critical normal stress used to produce deformation twins can be calculated as ${\sigma }_{T}={\tau }_{T}/0.5$, which is combined with Eq. (6), as expressed as follows:

7 $${\sigma }_{T}=\frac{4\lambda }{{b}_{p}}$$

As long as the tensile stress, *σ*_*d*_, overcomes the critical normal stress, *σ*_*T*_, the deformation twins in dendrites emerge. Combining Eqs (4) and (7), it is obtained that:

8 $${\sigma }_{d}\ge {\sigma }_{T}$$

The composite subjected to tensile loading produces a certain quantity of deformation twins, as can be observed in Fig. 5. And a large amount of dislocations and stacking faults are observed near the boundary of the twins. We simplify the process and evaluate the Burgers vector, *b*_*p*_, in Eq. (7) with the order of magnitudes (nanometer) into account, and the tensile stress of dendrites, *σ*_*d*_, is reached 1129 MPa, counted from Eq. (4) at the strain of 2.1%. As a consequence, the SFE, *λ*, can achieve 282 mJ/m^−2^ according to Eq. (7). The SFE is 150 mJ/m^−2^ in hcp Ti alloys^53^, 55 mJ/m^−2^ in hcp pure Mg^54^ and ranges from 60 to 100 mJ/m^−2^ in the NiX (Ti, Zr, Ta) systems^55^, which strongly demonstrates that the stress within dendrites has reached a critical stress to form the deformation twinning, and the twins indeed appear in present work.

The strain goes on with further deformation, and the glass matrixes yield, both of which indicate the coming of the plastic-plastic stage. The tensile stress of the composite is estimated to be^47^:

9 $$\sigma =\frac{1}{\sqrt{3}{c}_{m}}[\sqrt{3}{\sigma }_{ym}-3{E}_{m}(1-\beta )\frac{{\varepsilon }^{p}}{\sqrt{3}}]$$

Eq. (9) can be expressed as:

10 $$\sigma ={\sigma }_{ym}-\,0.524{E}_{m}{\varepsilon }^{p}$$

The glass matrix yields, and the following changing of the stress in the matrix makes it possible for the initiation of shear bands^56^. Normally, the glass matrix (phase) exhibits strain softening, accompanied by the occurrence of multiple shear bands after yielding^57^. Nevertheless, the density of shear bands is limited, as shown in Fig. 3(c), which can be regarded as the softening effect of the glass matrix defeated by work hardening induced by the TWIP effect in dendrites. Referring to the dendrites in stage (III), the stress in dendrites varies with increasing strain, and many deformation twins can be found, as shown in Fig. 5(a). The dislocation source becomes lacking because of the twinning barriers generated in stage (II). And subsistent twinning partials with different orientations can be regarded as deformation twinning^47^. The further explanation about the deformation twins is referred to dislocation reactions. The dislocations are difficult to move due to the hamper of deformation twins, and the perfect dislocations dissociate into partial dislocations due to the formation of stacking faults^58^. The effect of the dislocations forms new stacking faults and subsequently leads to new deformation twins with other orientations^59–64^. The formation of deformation twins in different stages contributes to work hardening of the composites. Shear bands produced within the glass matrix will result in a catastrophic fracture but not for dendrites. On the contrary, the high strength of the glass matrix provides enough stress to induce deformation twins in dendrites.

In accordance with the analysis, the deformation mechanism of the present MGMC upon tension consist of three parts, elastic-elastic, elastic–plastic, and plastic–plastic stages, which usually in accord with the deformation stages (I), (II), and (III), respectively. Within the stage (II) and (III), homogeneous elongation (that is work hardening) can be found, and accompanied by fracture without necking. Homogeneous elongation is extremely necessary for engineering materials subjected to tensile loading, on account of inhomogeneous deformation (that is necking) may reduce the service life of the engineering materials. Obviously, it is of importance for structural applications to appear work hardening in service. The TWIP effect that brings about a pronounced plastic deformation within dendrites promotes the work hardening along with the composite yields. And that, the glass matrix with high yield strength makes sure to achieve a high tensile strength of the composite.

## Conclusion

In summary, the *in-situ* metallic glass matrix composite (MGMC) Ti_43_Zr_32_Ni_6_Ta_5_Be_14_ (at %) displays an excellent mechanical performance upon tension at room temperature, achieving a high tensile strength of ~1470 MPa and a plastic strain of ~4%. Both tensile deformation micro-mechanisms and work-hardening behavior, which is induced by TWIP effects, are investigated by experimental and theoretical calculations in this work. The deformation twins found in dendrites acting as plasticity carriers are strongly demonstrated. The essential relations at different stages, the elastic–elastic, elastic–plastic, and plastic–plastic stages that usually matchup the elastic, work-hardening and the finally fractured deformation behaviors, are revealed by theoretical calculations. Work-hardening behavior is necessary for the materials as engineering materials, and the work hardening here is resulted from ductile dendrites and associated with homogeneous plastic deformation in the present composite. Moreover, the high yielding strength of the matrix makes contribution to the high strength of the composites. In consequence, the studies in our present work reveal that both deformation twining occurred within the *in-situ* crystalline dendrites and high yielding strength of the glass matrix ensure excellent mechanical properties of the composite, which provides a guide to design highly ductile MGMCs which exhibits remarkably homogeneous tensile elongation at room temperature.
